# Development and internal validation of diagnostic prediction models using machine-learning algorithms in dogs with hypothyroidism

**DOI:** 10.3389/fvets.2023.1292988

**Published:** 2023-12-19

**Authors:** Andrea Corsini, Francesco Lunetta, Fabrizio Alboni, Ignazio Drudi, Eugenio Faroni, Federico Fracassi

**Affiliations:** ^1^Department of Veterinary Medical Sciences, Alma Mater Studiorum-University of Bologna, Ozzano Emilia, Italy; ^2^Department of Veterinary Sciences, University of Parma, Parma, Italy; ^3^Department of Statistical Sciences, Alma Mater Studiorum-University of Bologna, Bologna, Italy

**Keywords:** diagnosis, canine, thyroid, thyroxine, endocrinology, logistic regression, machine learning, artificial intelligence

## Abstract

**Introduction:**

Hypothyroidism can be easily misdiagnosed in dogs, and prediction models can support clinical decision-making, avoiding unnecessary testing and treatment. The aim of this study is to develop and internally validate diagnostic prediction models for hypothyroidism in dogs by applying machine-learning algorithms.

**Methods:**

A single-institutional cross-sectional study was designed searching the electronic database of a Veterinary Teaching Hospital for dogs tested for hypothyroidism. Hypothyroidism was diagnosed based on suggestive clinical signs and thyroid function tests. Dogs were excluded if medical records were incomplete or a definitive diagnosis was lacking. Predictors identified after data processing were dermatological signs, alopecia, lethargy, hematocrit, serum concentrations of cholesterol, creatinine, total thyroxine (tT4), and thyrotropin (cTSH). Four models were created by combining clinical signs and clinicopathological variables expressed as quantitative (models 1 and 2) and qualitative variables (models 3 and 4). Models 2 and 4 included tT4 and cTSH, models 1 and 3 did not. Six different algorithms were applied to each model. Internal validation was performed using a 10-fold cross-validation. Apparent performance was evaluated by calculating the area under the receiver operating characteristic curve (AUROC).

**Results:**

Eighty-two hypothyroid and 233 euthyroid client-owned dogs were included. The best performing algorithms were naive Bayes in model 1 (AUROC = 0.85; 95% confidence interval [CI] = 0.83–0.86) and in model 2 (AUROC = 0.98; 95% CI = 0.97–0.99), logistic regression in model 3 (AUROC = 0.88; 95% CI = 0.86–0.89), and random forest in model 4 (AUROC = 0.99; 95% CI = 0.98–0.99). Positive predictive value was 0.76, 0.84, 0.93, and 0.97 in model 1, 2, 3, and 4, respectively. Negative predictive value was 0.89, 0.89, 0.99, and 0.99 in model 1, 2, 3, and 4, respectively.

**Discussion:**

Machine learning-based prediction models were accurate in predicting and quantifying the likelihood of hypothyroidism in dogs based on internal validation performed in a single-institution, but external validation is required to support the clinical applicability of these models.

## Introduction

Hypothyroidism is considered a common endocrine disease in dogs, although its actual prevalence remains unknown. The most typical clinical signs include dermatological abnormalities (e.g., alopecia, poor-quality coat, pyoderma, seborrhea) and non-specific metabolic signs (e.g., decreased appetite, weight gain, lethargy, asthenia, and cold intolerance) (1). The most common clinicopathological abnormalities are hypercholesterolemia and mild to moderate normocytic normochromic non-regenerative anemia. Hypothyroid dogs less commonly show azotemia, increased liver enzymes and creatine kinase activity, and increased serum fructosamine concentrations (1–4). The diagnosis is easily confirmed in dogs with a clinical suspicion of hypothyroidism and concurrent low serum total thyroxine (tT4) concentration and high serum thyroid-stimulating hormone (TSH) concentrations. However, serum tT4 can be decreased in euthyroid dogs due to non-thyroidal illness syndrome (NTIS) or previous/ongoing drug treatment (e.g., glucocorticoids, phenobarbital, sulphonamides, tyrosine kinase inhibitors) (5–10). Serum TSH can be normal in 20 to 40% of hypothyroid dogs (11, 12). Moreover, breed-specific reference intervals have been reported in different breeds (e.g., Greyhounds and other Sighthounds, Basenji, Dogue de Bordeaux) (13–16). For all these reasons, the correct assessment of thyroid function in dogs can often be challenging, possibly leading to overdiagnosis of hypothyroidism. The vast majority of hypothyroid dogs obtain a complete resolution of clinical signs and clinicopathological abnormalities with appropriate levothyroxine supplementation. On the contrary, euthyroid dogs misdiagnosed with hypothyroidism receive inappropriate treatment which fails to improve clinical signs and leads to a possible delay in achieving the correct diagnosis, unjustified costs for the owner, and the risk of iatrogenic hyperthyroidism. When in doubt, additional testing to confirm the diagnosis is a better approach than a therapeutic trial with levothyroxine. Free T4 measured by equilibrium dialysis has a higher specificity than tT4; however, it is laborious and expensive (11). A recombinant human TSH (rhTSH) stimulation test (TSHst) or a thyroid scintigraphy are currently considered to be the gold standards for confirming hypothyroidism; however, both are expensive for the owners and not readily available for primary care practitioners (4, 17–20). Recently, prediction models aimed to assist the diagnostic process have been formulated both in human and veterinary medicine, using regression or machine learning methods (21–23). A prediction model could improve the blood testing predictive values, thus supporting or discouraging the therapeutic choice, and suggesting when it is or is not appropriate to carry out more demanding tests, such as gold standard testing. This approach could improve the overall diagnostic accuracy and decrease the rate of misdiagnosis. To be clinically useful in primary care practice, an easy-to-use tool must be developed from a model.

The aim of this study was to develop an easy-to-use prediction tool to assist in clinical decision-making when evaluating dogs with suspected hypothyroidism. It was hypothesized that a machine learning-based model built on clinical signs and clinicopathological data would help in defining the likelihood of hypothyroidism in dogs in which it was suspected.

## Materials and methods

### Study design

The digital patient management system (Fenice, Zaksoft Software Technology) of the Veterinary Teaching Hospital of the University of Bologna was searched for dogs in which a joint measurement of basal tT4 and endogenous TSH serum concentrations, or an rhTSH stimulation test were available in the period between January 2006 and September 2020. The medical records of the hospital were searched for information regarding signalment (i.e., sex, age, and breed), body weight, body condition score (BCS), presence/absence of clinical signs (i.e., asthenia, lethargy, polyuria/polydipsia, changes in appetite, obesity, alopecia, dermatological signs, and neurological alterations), complete blood count (i.e., hematocrit value [HCT], hemoglobin concentration [Hgb], red blood cell [RBC] count, mean corpuscular volume [MCV] and mean corpuscular hemoglobin concentration [MCHC]), serum chemistry (i.e., cholesterol, triglycerides, and creatinine concentrations, alanine aminotransferase [ALT], and aspartate aminotransferase [AST] activities), urinalysis (specific gravity and urine protein to creatinine ratio), and thyroid function evaluation (i.e., basal and post-rhTSH stimulation serum tT4 concentrations and TSH concentrations). The category of dermatological signs included all dermatological abnormalities other than alopecia, such as dry/poor quality coat, skin hyperpigmentation, pyoderma, seborrhea and recurrent otitis. When available, a diagnosis and follow-up of the patients were obtained. Dogs were excluded from the study based on the following criteria: (a) complete lack of anamnestic, clinical or clinicopathological data, (b) thyroid function testing carried out for research purposes or to monitor hypothyroid dogs receiving treatment, (c) the presence of congenital or suspect secondary hypothyroidism, and (d) treatment with levothyroxine in the month before the hormonal tests. Congenital hypothyroidism was defined based on age of presentation and typical clinical signs, while secondary hypothyroidism was suspected in dogs with acquired hypothyroidism and concurrent pituitary macrotumor. Dogs with concurrent diseases and dogs that were receiving medications known to affect serum tT4 and TSH concentration were included, providing their diagnosis was based on rhTSH stimulation results. Cases in which a clear confirmation or exclusion of hypothyroidism could not be obtained were also excluded from the study. Dogs were classified as euthyroid based on a serum post-rhTSH stimulation tT4 > 1.7 μg/dL or if they showed (a) a normal tT4 and TSH serum concentration and (b) lack of clinical or clinicopathological abnormalities consistent with hypothyroidism, or a diagnosis other than hypothyroidism was reached, or if clinical signs improved without treatment with levothyroxine. Dogs were classified as hypothyroid based on a serum post-TSH stimulation tT4 < 1.3 μg/dL or if they showed an increased TSH serum concentration associated with a decreased basal tT4 serum concentration, with clinical or clinicopathological signs consistent with hypothyroidism (4). Two authors (AC, FF) reviewed all the case records.

### Clinicopathological analysis

All the analysis was performed in the Clinical Pathology Laboratory of our Institution. Fasting blood samples collected for biochemistry analysis and for hormonal assays were processed on a routine basis, according to quality standard procedures. Urine samples were prepared using low-speed centrifugation. The urine supernatant was separated and specific gravity was analyzed using a spectrometer. The serum and urine chemistry analysis were carried out using two different analyzers, which replaced each other during the inclusion period: Olympus AU400 (Two Corporate Center Drive, Melville, New York, United States) from 2006 to 2016, and Beckman Coulter-Olympus AU 480 (Brea, California, United States) from 2016 to 2020; both analyzers used the same methods for serum and urine chemistry analysis. Blood samples for the complete blood count were collected in K_3_ ethylene diamine tetra-acetic acid (EDTA) tubes and analyzed using two different analyzers, which replaced each other during the inclusion period: Abbott Cell-Dyn 3,500 (Abbott Laboratories, Green Oaks, Illinois, United States) from 2006 to 2010, and ADVIA 2120 (Siemens Healthcare Diagnostics, Tarrytown, New York, United States) from 2010 to 2020. Hormonal analysis on the serum was carried out using two different analyzers, which replaced each other during the inclusion period: Immulite One (Medical Systems SpA, Genova, Italy) from 2006 to 2017, and Immulite 2000 (Siemens Healthineers, Flanders, New Jersey, United States) from 2017 to 2020. The serum tT4 concentration was determined using commercially available chemiluminescent enzyme immunometric assays (Immulite Canine Total T4, Diagnostic Products Corporation; Immulite 2000 Canine Total T4, Diagnostic Products Corporation, Los Angeles, California, United States) validated for use in dogs (reference range 1 to 3.98 μg/dL [12.8 to 51.2 nmol/L]) (24, 25). The serum TSH concentrations were measured using chemiluminescent enzyme immunometric assays (Immulite Canine TSH, Diagnostic Products Corporation; Immulite 2000 Canine TSH, Diagnostic Products Corporation, Los Angeles, California, United States) validated for use in dogs (upper limit of the reference range 0.38 ng/mL) (26). Based on product data sheets, both the method comparison between Immulite 2000 canine total T4 and Immulite One canine total T4, and the method comparison between Immulite 2000 canine TSH and Immulite One canine TSH showed strong correlations (R = 0.991 and R = 0.988, respectively) (PIL2KCT-5 Immulite 2000 Canine Total T4 [June 27, 2005]; PIL2KKT-15 Immulite 2000 Canine TSH [March 6, 2017]). The results of the biochemistry analysis, the hormonal assays, and the complete blood count were compared with reference intervals calculated internally in the Clinical Pathology Laboratory, according to previously published guidelines (27). The TSH stimulation test was carried out using a dose of 75 μg/dog of rhTSH (Thyrogen, Genzyme Corporation, Suffolk, UK) (4). Blood samples were collected immediately before and 6 h after the intravenous administration of rhTSH.

### Statistical analysis

Machine learning models were built to describe how the variability of the response variable (i.e., hypothyroidism, no hypothyroidism) was generated by the relationship with the explanatory variables (i.e., clinical and clinicopathological parameters) using an algorithm approach.

The dataset obtained from the medical records was analyzed to identify and exclude variables which had >5% of missing data. Then, feature selection was carried out to remove irrelevant and redundant information, and define a subset of variables which would provide good prediction results. The feature selection was conducted in two phases. In the first phase, the significantly related variables were identified by means of an analysis of mutual dependence and the variables which had a significant relationship with the objective variable were identified using a filter method, such as mutual information (MI); a permutation test was applied to the MI to verify its significance. Based on these results, 4 different models, which included different combinations of variables, were created. Specifically, the models differed based on the inclusion of quantitative or qualitative variables and the inclusion or exclusion of thyroid hormones concentrations. Model 1 and 2 were built using quantitative variables, without and with thyroid hormones concentrations, respectively; Model 3 and 4 were built using qualitative variables, without and with thyroid hormones concentrations, respectively. In the qualitative models, the categories were defined as normal if the value was within the reference interval; increased if the value was above the RI; decreased if the value was below the RI; markedly increased (cholesterol) if the value was 2 times higher than the upper reference limit and markedly decreased (tT4) if the value was below the lower limit of detection of the assay (6 mmol/L). In the second phase, a Wrapper method, in which the influence of the learning algorithm was considered, was applied to these 4 models. Six different learning algorithms, namely Classification Tree (CT), Random Forest (RF), Gradient Boosting Machine (GBM), Support Vector Machine (SVM), Logistic Regression (LR) and naive Bayes (NB), were applied to cover a broad spectrum of existing tools. The evaluation of the predictive performance was performed on the original dataset in a 10-fold cross-validation setup (i.e., the initial sample was randomly divided into 10 sub-samples and, from time to time, each of these 10 sub-samples was used as a validation set as compared to the other 9 sub-samples, used as training sets), repeated 10 times, to prevent the results from being affected by the selection of the training and test samples. For each setup, the set of classification rules which best predicted the data of the training set was created; then, the classification rules were applied to the test set and, finally, within the test set, the predicted values were compared with the real observed values, and the performance of the model was evaluated by calculating the normalized Matthews correlation coefficient (nMCC). The nMCC ranges from 0 to 1, with 0 indicating perfect misclassification, 0.5 indicating random classification, and 1 indicating perfect classification (28). The predictive performance estimated by this process was defined as the apparent performance because it was calculated on the same dataset used to develop the prediction model. For each prediction model, the optimal model was defined as the one with the highest apparent performance, defined as the highest nMCC. The sensitivity, specificity, positive predictive values (PPV), negative predictive values (NPV), accuracy, and area under the receiver operating characteristic curve (AUROC) of the optimal models were reported. The statistical analysis was carried out using Cran-R software, and the statistical significance was set at *p* < 0.05.

### User interface building-up

A graphic user interface was created to facilitate the implementation of the prediction models in clinical practice. In order to make the application more user-friendly, an attempt was made to standardize the interface by identifying what was the common combination of variables of the different optimal models which presented the best predictive capacity, and then verifying whether these modified models were significantly different from the optimal ones. The nMCC of the optimal and the modified models were compared using t-test.

## Results

### Dataset

The medical records of 619 dogs were recovered from the digital patient management system; of these, 304 (49%) cases presented at least one exclusion criterion. Three hundred and fifteen (51%) cases were ultimately included in the study. Eighty-two (26%) dogs were classified as hypothyroid and 233 (74%) were classified as euthyroid.

In the hypothyroid group, there were 40 (49%) males, of which 6 were neutered, and 42 (51%) females, of which 28 were spayed. The median age was 8.0 years (range, 1.8–15.6), and the median body weight was 30.0 kg (range, 7.3–69.0). The most represented breeds were mixed breeds (*n* = 26; 32%), Dobermann Pinschers (*n* = 7; 9%), and English Setters (*n* = 5; 6%). Serum TSH concentration was within reference interval in 20 (24%) hypothyroid dogs, all of which were diagnosed based on rhTSHst. Overall, in 39 (48%) cases an rhTSHst was carried out to confirm the clinical suspicion of hypothyroidism.

In the euthyroid group, there were 123 (53%) males, 31 of which were neutered, and 110 (47%) females, of which 66 were spayed. The median age was 9.2 years (range, 1.3–17.3), and the median body weight was 25.5 kg (range, 1.4–84.0). Most represented breeds were mixed breeds (*n* = 59; 25%), Labrador Retrievers (n = 22; 9%), Dobermann Pinschers (*n* = 18; 8%), and Golden Retrievers (*n* = 13; 6%). In 67 (29%) cases, a TSHst was required to exclude hypothyroidism due to a clinical (*n* = 45; 67%), clinicopathological (*n* = 12; 18%), or clinical and clinicopathological (*n* = 10; 15%) suspicion of the disease.

For both groups, the results of quantitative and qualitative variables are listed in Tables 1, 2, respectively. Concurrent diseases were overall reported in 198/315 (63%) dogs, 21 (26%) hypothyroid and 177 (76%) euthyroid dogs.

**Table 1:** Descriptive statistics for quantitative variables in hypothyroid dogs (*n* = 82) and euthyroid dogs (*n* = 233).

Variables		Hypothyroid	Euthyroid	Reference interval
		Median (range)	Median (range)	
Age (years)		8.0 (1.8–15.6)	9.2 (1.3–17.3)	
Body weight (kg)		30 (7.3–69)	25.5 (1.4–84)	
tT4	nmol/L	6.4 (1.3–14.6)	19.5 (3.5–51.9)	13–51
	μg/dl	0.5 (0.1–1-1)	1.5 (0.3–4-0)	1.0–4.0
TSH (ng/ml)		0.7 (0.0–8.9)	0.1 (0.0–1.5)	0.03–0.38
Post-stim tT4	nmol/L	6.4 (1.3–20.6)	37.1 (9.5–126.0)	
	μg/dl	0.5 (0.1–1.6)	2.9 (0.7–9.8)	
Cholesterol (mg/dl)		554 (140–2025)	274 (76–1,030)	123–345
Creatinine (mg/dl)		1.1 (0.5–2.0)	0.9 (0.4–6.3)	0.75–1.4
ALT (U/L)		68 (10–980)	59 (17–1,285)	15–65
HCT (%)		38.6 (28.7–51.6)	46.2 (19.8–60.7)	39–58
Hemoglobin (gr/dl)		13.0 (9.6–18.0)	15.7 (6.5–21.1)	14–19
Red blood cell count (x10^6^/mm^3^)		5.79 (4.28–8.25)	6.79 (4.13–9.42)	5.65–8.40
MCV (fL)		68.0 (56.9–82.0)	68 (54.8–79.7)	63–77
MCHC (gr%)		33.6 (21.2–36.7)	33.8 (24.4–41.3)	32–37

tT4, total thyroxine; TSH, thyroid-stimulating hormone; ALT, alanine-amino transferase; AST, aspartate-amino transferase; HCT, hematocrit; MCV, mean corpuscular volume; MCHC, mean cellular hemoglobin concentration. Number of missing data is reported in Supplementary Table S1.

**Table 2:** Descriptive statistics for qualitative variables in hypothyroid dogs (n = 82) and euthyroid dogs (*n* = 233).

	Hypothyroid (*n* = 82)	Euthyroid (*n* = 233)
	Cases (%)	Cases (%)
Sex (male)	40 (49%)	123 (53%)
Sex (female)	42 (51%)	110 (47%)
Mixed breed	26 (32%)	59 (25%)
Asthenia	45 (55%)	83 (36%)
Lethargy/Depression	48 (59%)	58 (25%)
Polyuria/Polydipsia	9 (11%)	44 (19%)
Decreased appetite	8 (10%)	23 (10%)
Obesity	32 (39%)	43 (18%)
Alopecia	49 (60%)	70 (30%)
Dermatitis	28 (34%)	44 (19%)
Neurological abnormalities	17 (21%)	71 (30%)

### Statistical model

**Figure 1:**
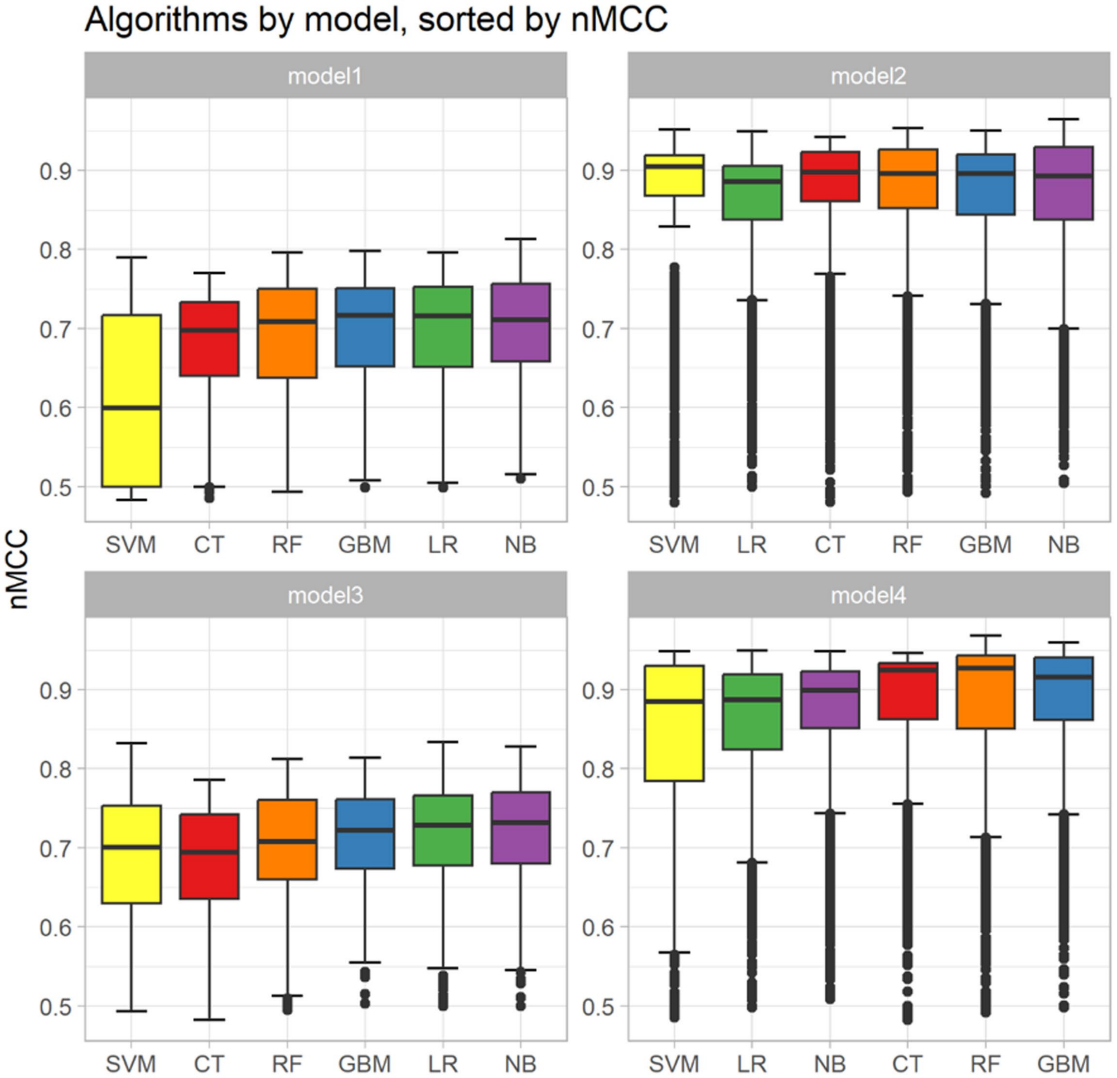
Box and whisker plots showing the apparent performances of the 4 models. For each model, the predictive performance of the 6 machine-learning algorithms (i.e., Classification Tree [CT], Random Forest [RF], Gradient Boosting Machine [GBM], Support Vector Machine [SVM], Logistic Regression [LR] and naïve Bayers [NB] were sorted using a normalized Matthews Correlation Coefficient (nMCC). The boxes represent the interquartile range from the 25th to the 75th percentile. The horizontal bar in each box represents the median value. The whiskers represent the interquartile range from the 2.5th to the 97.5th percentile, with the outliers represented as dots. The dotted lines represent the limits of the reference interval.

Analysis of the missing data is reported in Supplementary Table S1. The result of the analysis of the correlation performed between the continuous variables is represented in Supplementary Table S2. Based on this, the Hgb and RBC counts were removed because of the overlapping with HCT. The results of the feature selection identified 11 variables: breed, HCT, serum concentrations of cholesterol, creatinine, serum tT4 and TSH concentrations, and the presence of alopecia, dermatopathy, lethargy/depression, asthenia, and/or obesity (Table 3). These variables were grouped into 4 different models, as previously described (Table 4). The results of the performance evaluation for the 4 different models are reported in Figure 1. Based on the values of the nMCC, the best-performing algorithms were NB for models 1 and 2, LR for model 3 and RF for model 4 (Table 5). The sensitivity, specificity, PPV, NPV, accuracy, and AUROC of the optimal models were described in Table 6.

**Table 3:** Feature selection using mutual information.

Variable	MI	*p* value	Significance
tT4	0.596	<0.001	***
TSH	0.0895	<0.001	***
Cholesterol	0.166	<0.001	***
Hgb	0.126	<0.001	***
Hct	0.139	<0.001	***
RBC	0.129	<0.001	***
Lethargy/depression	0.0659	<0.001	***
Alopecia	0.0456	<0.001	***
Asthenia	0.0279	0.001	**
Obesity	0.0253	0.002	**
Dermatopathy	0.0167	0.005	**
Breed	0.0571	0.013	*
Creatinine	0.0182	0.024	*
Neurologic alterations	0.00711	0.08	
Polyuria/polydipsia	0.00637	0.118	
Age	0.0191	0.133	
Appetite	0.00805	0.208	
MCHC	0.0161	0.281	
ALT	0.0142	0.294	
Weight	0.0117	0.374	
Sex	0.00762	0.385	
MCV	0.00306	0.892	

MI, mutual information; tT4, total thyroxine; TSH, thyroid-stimulating hormone; Hgb, hemoglobin; Hct, hematocrit; RBC, red blood cells; MCHC, mean corpuscular hemoglobin concentration; ALT, alanine-amino transferase MCV, mean corpuscular volume.

**Table 4:** List of variables considered for inclusion in the four different models.

**Model**	**Variables**
model1	Cholesterol (normal/increased/markedly increased), Hct (decreased/normal), creatinine (normal/increased), asthenia (yes/no), lethargy/depression (yes/no), alopecia (yes/no), obesity (yes/no), dermatopathy, breed
model2	tT4 (normal/decreased/markedly decreased), TSH (normal/increased), cholesterol (normal/increased/ markedly increased), Hct (decreased/normal), creatinine (normal/increased), asthenia (yes/no), lethargy/depression (yes/no), alopecia (yes/no), obesity (yes/no), dermatopathy (yes/no), breed
model3	Cholesterol (mg/dL), Hct (%), creatinine (mg/dL), asthenia (yes/no), lethargy/depression (yes/no), alopecia (yes/no), obesity (yes/no), dermatopathy (yes/no), breed
model4	tT4 (nmol/L), TSH (ng/mL), cholesterol (mg/dL), Hct (%), creatinine (mg/dL), asthenia (yes/no), lethargy/depression (yes/no), alopecia (yes/no), obesity (yes/no), dermatopathy (yes/no), breed

Hct, hematocrit; tT4, total thyroxine; TSH, thyroid-stimulating hormone.

Models 1 and 2 were defined as qualitative models. Models 3 and 4 were defined as quantitative models. In the qualitative models, the categories were defined as normal if the value was within the reference interval (RI); increased if the value was above the RI; decreased if the value was below the RI; markedly increased (cholesterol) if the value was 2 times higher than the upper reference limit and markedly decreased (tT4) if the value was below the lower limit of detection of the assay (6 mmol/L).

**Table 5:** Comparison between the explanatory variables and the predictive performances of the best performing machine learning models (optimal models) and of the machine learning models selected for implementation in the user-friendly prediction tool (modified models).

Model group	Model		MLA	Variables included	nMCC	t-test
					Mean (95% CI)	*P* value
No hormone test	*Model1*	optimal	NB	Cholesterol,Hct,Creatinine,Lethargy_Depression,Alopecia	0.81 (0.80–0.83)	
		**modified**	**RF**	**Cholesterol,Hct,Asthenia,Lethargy_Depression,Alopecia**	0.80 (0.78–0.81)	0.1535
	*Model3*	optimal	LR	Cholesterol,Hct,Lethargy_Depression,Alopecia	0.83 (0.82–0.85)	
		**modified**	**LR**	**Cholesterol,Hct,Asthenia,Lethargy_Depression,Alopecia**	0.82 (0.80–0.84)	0.3089
With hormone test	*Model2*	optimal	NB	tT4,TSH,Cholesterol,Hct,Creatinine,Lethargy_Depression	0.96 (0.96–0.97)	
		**modified**	**NB**	**tT4,TSH,Cholesterol,Hct,Creatinine,Ashtenia**	0.96 (0.95–0.96)	0.1312
	*Model4*	optimal	RF	tT4,TSH,Hct,Creatinine,Dermatopathy	0.97 (0.96–0.98)	
		**modified**	**RF**	**tT4,TSH,Cholesterol,Hct,Creatinine,Asthenia**	0.96 (0.95–0.97)	0.083

MLA, machine learning algorithm; NB, Naïve-Bayes; LR, Logistic Regression; RF, Random Forest; Hct, hematocrit; tT4, total thyroxine; TSH, thyroid-stimulating hormone; nMCC, normalized Matthews Correlation Coefficient; CI, confidence interval.

The machine learning models selected for implementation in the user-friendly prediction tool (modified models) are reported in bold.

**Table 6:** Main indicators of the predictive performance of the best performing machine learning models (optimal models) and machine learning models selected for implementation in the user-friendly prediction tool (modified models).

**Model**		**Accuracy**	**Sensitivity**	**Specificity**	**PPV**	**NPV**	**AUROC**
*Model1*	optimal	0.86 (0.84–0.87)	0.70 (0.67–0.73)	0.92 (0.90–0.93)	0.76 (0.72–0.79)	0.89 (0.88–0.90)	0.85 (0.83–0.86)
	**modified**	0.85 (0.83–0.86)	0.60 (0.56–0.63)	0.94 (0.93–0.95)	0.79 (0.76–0.83)	0.86 (0.85–0.88)	0.84 (0.83–0.86)
*Model3*	optimal	0.87 (0.86–0.89)	0.68 (0.64–0.71)	0.95 (0.94–0.95)	0.84 (0.81–0.86)	0.89 (0.87–0.90)	0.88 (0.86–0.89)
	**modified**	0.87 (0.86–0.88)	0.66 (0.62–0.70)	0.94 (0.94–0.95)	0.81 (0.78–0.84)	0.88 (0.87–0.90)	0.87 (0.86–0.89)
*Model2*	optimal	0.97 (0.97–0.98)	0.97 (0.96–0.98)	0.97 (0.96–0.98)	0.93 (0.91–0.94)	0.99 (0.98–0.99)	0.98 (0.97–0.99)
	**modified**	0.96 (0.96–0.97)	0.96 (0.95–0.97)	0.97 (0.96–0.97)	0.92 (0.90–0.93)	0.98 (0.98–0.99)	0.98 (0.97–0.99)
*Model4*	optimal	0.97 (0.97–0.98)	0.94 (0.92–0.95)	0.99 (0.98–0.99)	0.97 (0.96–0.99)	0.98 (0.97–0.98)	0.99 (0.98–0.99)
	**modified**	0.97 (0.96–0.97)	0.94 (0.92–0.96)	0.98 (0.97–0.98)	0.94 (0.93–0.96)	0.98 (0.97–0.98)	0.98 (0.98–0.99)

Results are reported as mean (95% confidence interval).

AUROC, area under the receiver operating characteristic curve; PPV, positive predictive value; NPV, negative predictive value.

### User interface

**Figure 2:**
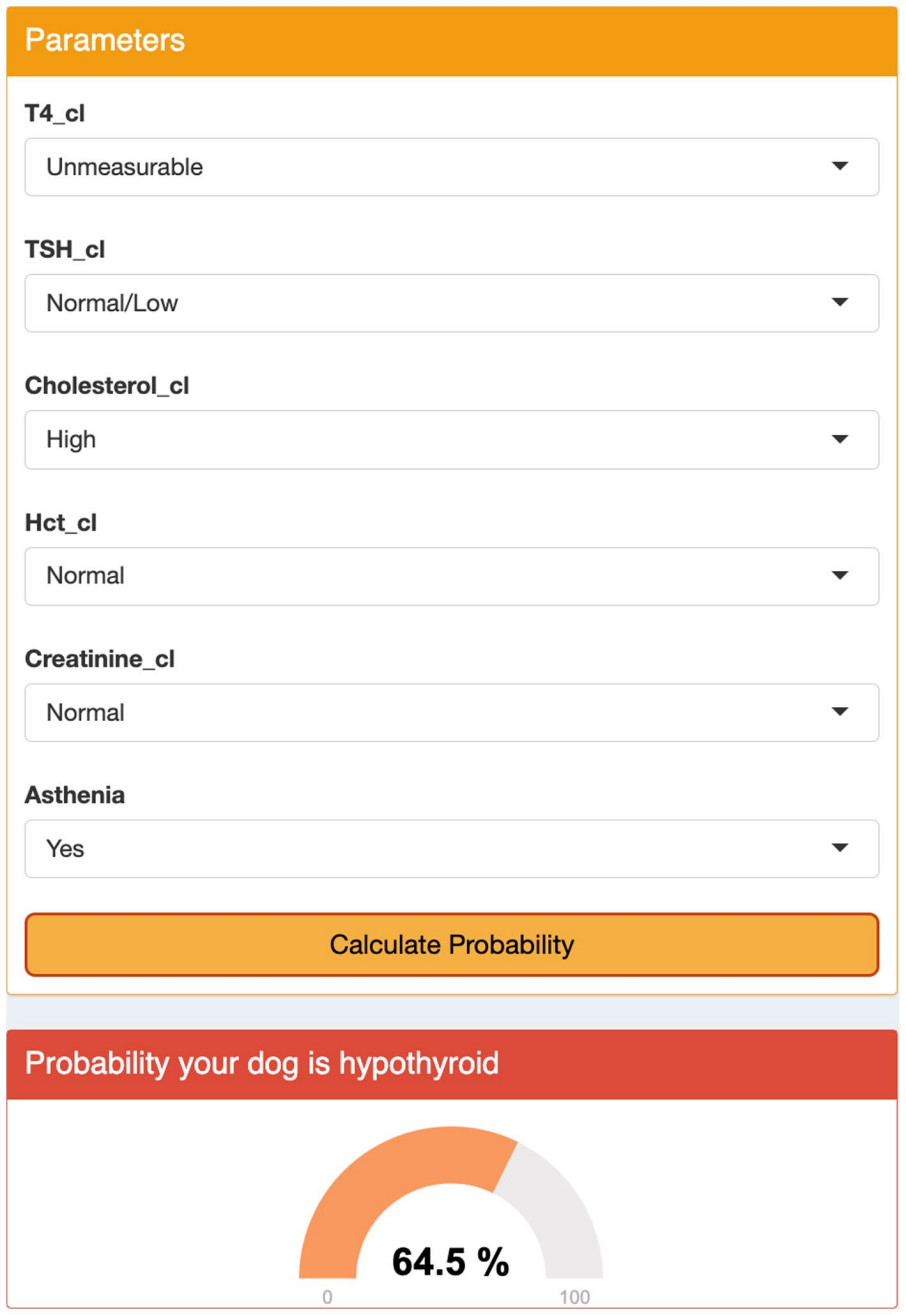
Graphical user interface for Model 3. The user selects the appropriate category for each parameter from the drop-down menus and clicks the ‘Calculate Probability’ button. The algorithm displays the probability of the dog being hypothyroid as a percentage.

**Figure 3:**
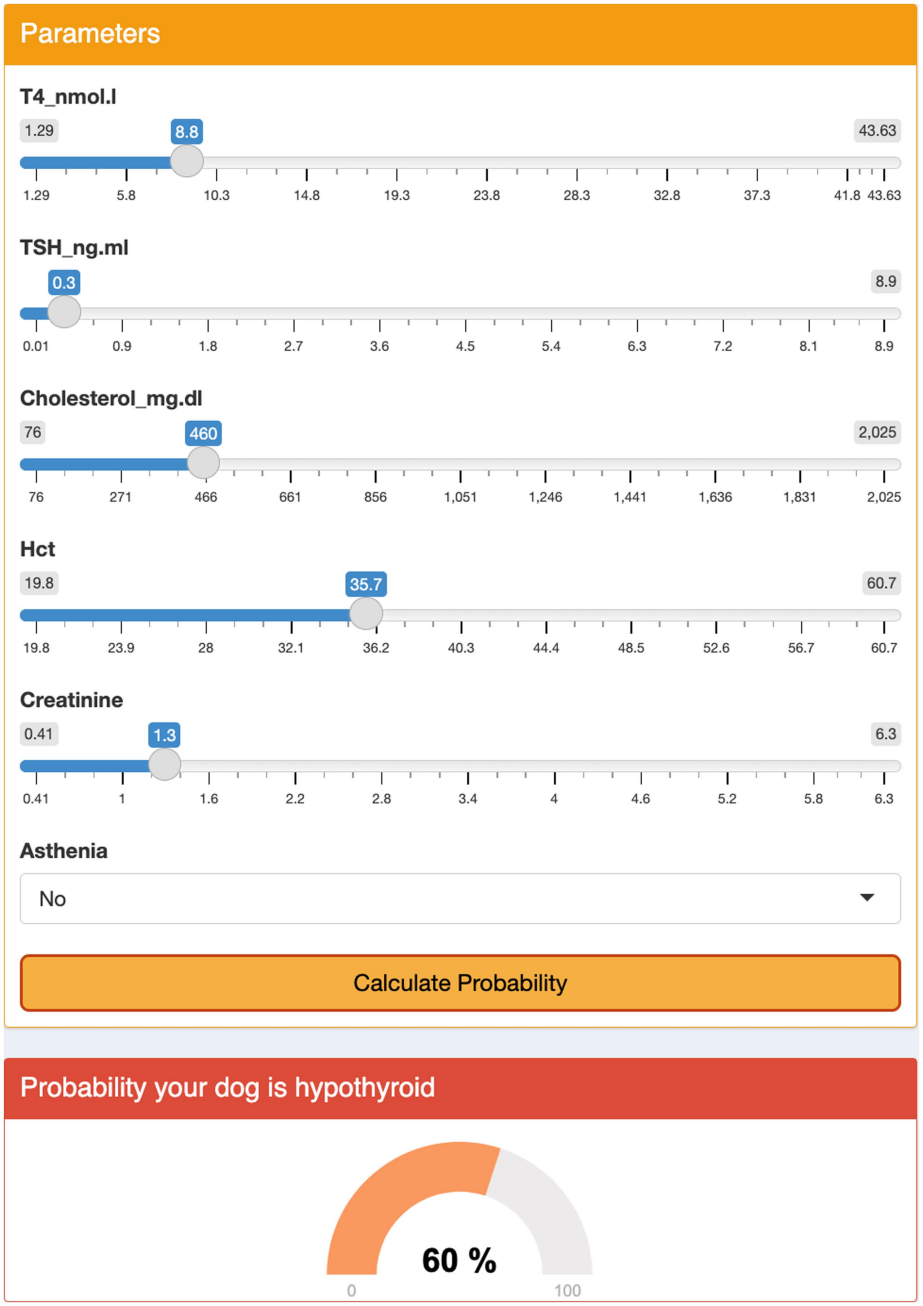
Graphical user interface for model 4. The user enters medical record numbers using the menu and clicks the ‘Calculate Probability’ button. The algorithm displays the probability of the dog being hypothyroid as a percentage.

The modified models, which included a set of variables common among different models, did not show a significant loss in predictive ability as compared to the optimal models (Table 5). The interface was designed using a sequential approach. First, the veterinarian had to select the preferred model; then, the clinical and clinicopathological variables required could be added to the model using input boxes. At that point, the interface displayed a percent chance that the patient had hypothyroidism (Figures 2, 3).

## Discussion

The Authors demonstrated that the prediction models described in the present study, based on clinical and clinicopathological parameters obtained from a single-institution and built using the algorithm modeling approach, have good accuracy in determining whether a dog is hypothyroid or not. A graphic user interface was also created which translated the results of these models into a predicted likelihood of the disease, thus enabling their application in clinical practice, as long as external validation supports the diagnostic accuracy described herein.

Estimating the probability of a disease is inherently a multivariable-based process; every clinician naturally integrates signalment, clinical signs and test results to assess the probability of disease which can very rarely be defined on the basis of a single predictor. A multivariable diagnostic prediction model is a mathematical equation which relates multiple predictors for a particular individual with the probability of the presence (diagnosis) of a particular outcome (29, 30). There are no validated diagnostic prediction models for hypothyroidism in dogs; however, they could prove to be extremely useful, considering that hypothyroidism can be difficult to diagnose especially when concurrent diseases are present, serum TSH concentrations are not increased, and gold-standard testing (i.e., rhTSH stimulation test, thyroid scintigraphy) is not available or possible. Several studies have applied machine learning to create diagnostic prediction models in dogs and cats for different diseases, including kidney diseases in cats, canine leishmaniasis and endocrine diseases, such as hypoadrenocorticism and Cushing’s Syndrome in dogs (21, 22, 31–33). All the models presented in these studies were, for the most part, built on clinical data and the results of easy-to-perform laboratory testing results with good predictive performance. The predictors assessed in this study were identified *a priori* using the current knowledge of the disease, based on the existing scientific literature. All the predictors were demographic factors, clinical signs or clinicopathological data commonly assessed in dogs with suspected hypothyroidism which were easy and affordable to obtain in a clinical setting. The 9 predictors included in the final models were: alopecia, dermatological signs, lethargy/depression, asthenia, HCT, serum cholesterol concentration, and serum creatinine concentration plus serum tT4 and TSH concentrations. These results were consistent with the existing literature. Alopecia, other dermatological signs and lethargy are clinical signs commonly reported in hypothyroid dogs. None of them is specific; however, taken together, they are fairly indicative since the majority of hypothyroid dogs show at least one of them (2, 4, 34). Hypercholesterolemia and mild to moderate normocytic normochromic non-regenerative anemia are the most common clinicopathological abnormalities in hypothyroid dogs. Hypercholesterolemia, caused by an altered lipid metabolism, is reported in 70–80% of cases, and it has been suggested that the larger the increase in cholesterol the more likely hypothyroidism rather than non-thyroidal illness (2, 4, 34). Anemia is less common but still described in approximately 30–40% of cases, presumably as a consequence of the decreased production of erythropoietin (2, 4, 34). Azotemia results from decreased glomerular filtration rate due to lack of thyroid hormones and has been variably reported in hypothyroid dogs; the majority of studies have reported azotemia in approximately 10–15% of cases; however, it reached up to 33% in a recent study (1, 3, 4).

All four models presented in the present study showed good to excellent apparent performance in predicting the presence of hypothyroidism. The term apparent performance defined the predictive ability of a model quantified on the same data from which the model was built; it could result in an optimistic estimate of performance, due to overfitting. For this reason, an internal validation is necessary to adjust the model for overfitting which was carried out using a 10-fold cross-validation.

Based on the inclusion criteria applied (i.e., measurement of thyroid hormones), the original dataset, included for the most part, but not only, dogs in which hypothyroidism was at least deemed possible by the Authors or by the referring veterinarian. If dogs in which hypothyroidism was not suspected (i.e., sick dogs without clinical signs or clinicopathological abnormalities suggestive of hypothyroidism) were included, it is likely that the predictive performance of the present models would have been different. However, to optimize the predictive performance and its clinical utility, the model must be developed using a sample representative of the target population, namely dogs in which hypothyroidism is suspected; in fact, it is senseless and misleading to apply the prediction models if hypothyroidism is not even suspected at first.

As expected, the performance of the prediction models markedly improved when tT4 and TSH concentrations were included in the model. Even if models 1 and 3 were the least performing since they did not include tT4 and TSH, they were important since they could be applied to dogs when only clinical findings and a minimum clinicopathological database were available, and before thyroid hormone evaluation, thus helping in the assessment of the pre-test probability. Moreover, the models were built including both continuous variables (models 1 and 2) and mixed continuous and categorical variables (models 3 and 4). The continuous variables allowed for a fine-tuned assessment of the variable of interest; however, the reference interval for any variable usually depends on the laboratory which carries out the analysis. Thus, models 1 and 2 which incorporated categorical variables could be better suited for application in clinical practice, considering that the reference intervals for specific variables (e.g., serum cholesterol concentration) could vary between different centers, sometimes greatly, and that the reference intervals reported in the present study are not universally valid.

The prediction models presented in this study were neither meant to be used as substitutes for clinical reasoning nor as a gold-standard for diagnosis, but rather to support clinicians in their decision-making, helping them to define the likelihood of disease in an individual dog. In addition, the present prediction tool could be helpful in everyday practice in many different ways. At the time of the first consultation, the tool could give the clinicians a pre-test probability helping them to decide whether additional testing (e.g., serum tT4 and TSH measurement) was warranted or not. Even more, if thyroid testing is subsequently carried out, the tool would quantify the actual predictive value of the results obtained. A classic example is the approach to tT4 assessment in a dog with low pre-test-probability (e.g., no alopecia, no dermatological signs, no lethargy, no anemia and no hypercholesterolemia); in fact, mildly/moderately decreased tT4 has a low positive predictive value in this setting and, at the least, additional testing is required before starting treatment. On the contrary, if the dog had a high pre-test probability, moderately decreased tT4 could be enough to merit treatment with levothyroxine. Thus, obtaining a quantitative value of the predicted likelihood of hypothyroidism could aid in avoiding a misdiagnosis and help communication with the owners regarding the reasons behind the decision to discard hypothyroidism, start treatment, or suggest gold-standard testing.

Of note, any prediction model, even if proven accurate, should be trusted and routinely applied in clinical practice only if built on solid data and, as much as possible, free from bias. For this reason, some limitations should be assessed. The main limitation of the present study was the number of cases included; in fact, larger sample sizes would lead to the development of more robust prediction models. A general rule of thumb for the sample size required is to include 10 events (i.e., hypothyroid dogs) for each candidate predictor variable considered in the model (35). Based on this, the ideal sample for this study should have included approximately 200 hypothyroid dogs. However, models including less than 10 events per parameter should not be systemically disregarded and could still be clinically useful, especially if based on strong predictors, as is the case of the present models (36). The Authors carried out a case by case review of the medical records, and stringent inclusion criteria were applied to the original dataset. This approach was appropriate in order to minimize the risk of misclassification bias; however, some hypothyroid dogs were likely excluded from the study due to the inability to confirm the diagnosis. Furthermore, specific clinical signs not clearly stated as ‘present’ or ‘not present’ in the medical records were recorded as ‘not present’ in the present dataset, based on the assumption that clinicians usually do not record absent clinical signs. It is possible that some clinical signs remained unnoticed by the owners and were misclassified by the attending clinician; however, this was likely the same in both groups. The use of tT4 and TSH concentrations as part of the inclusion criteria may have overestimated the performance of model 2 and 4, but hormone concentrations were not used as sole criteria for classifying the dogs and, most importantly, the algorithm was designed to specifically consider the degree of serum tT4 decrease and serum TSH elevation. Finally, before applying these results to very different populations, an external validation should be performed. The Authors aim to carry out an external validation of their models, which can be done either by using independent data collected by the same authors but sampled from a later period of time or by using data collected by different investigators in different clinical settings (i.e., primary vs. secondary care) or different centers. This approach strongly supports the clinical applicability of prediction models and allows for updating and adjustments in the case that poor performance is detected (29, 30).

## Data availability statement

The raw data supporting the conclusions of this article will be made available by the authors, without undue reservation.

## Ethics statement

Ethical approval was not required for the studies involving animals in accordance with the local legislation and institutional requirements because it is not required by the local animal ethicals for studies using medical records and retrospectively collected data. No procedure was performed on animals for this study. Written informed consent was not obtained from the owners for the participation of their animals in this study because it is not required by the local animal ethicals for studies using medical records and retrospectively collected data. No procedure was performed on animals for this study.

## Author contributions

AC: Conceptualization, Data curation, Formal analysis, Investigation, Methodology, Project administration, Resources, Supervision, Validation, Visualization, Writing – original draft, Writing – review & editing. FL: Conceptualization, Data curation, Formal analysis, Investigation, Resources, Visualization, Writing – original draft, Writing – review & editing. FA: Data curation, Formal analysis, Investigation, Methodology, Software, Writing – original draft, Writing – review & editing. ID: Formal analysis, Methodology, Software, Supervision, Writing – original draft. EF: Data curation, Formal analysis, Investigation, Visualization, Writing – original draft, Writing – review & editing. FF: Conceptualization, Investigation, Methodology, Project administration, Resources, Supervision, Validation, Visualization, Writing – original draft, Writing – review & editing.

## Figures and Tables

**Table:** 

ALT	alanine aminotransferase
AST	aspartate aminotransferase
AUROC	area under the receiver operating characteristic curve
BCS	body condition score
CT	classification tree
EDTA	ethylene diamine tetre-acetic acid
GBM	gradient boosting machine
HCT	hematocrit value
Hgb	hemoglobin concentration
LR	logistic regression
MCHC	mean corpuscular hemoglobin concentration
MCV	mean corpuscular volume
MI	mutual information
NB	naïve Bayes
nMCC	normalized Matthews correlation coefficient
NPV	negative predictive value
NTIS	non-thyroidal illness syndrome
PPV	positive predictive value
RF	random forest
RBC	red blood cells
rhTSH	recombinant human thyroid-stimulating hormone
SVM	support vector machine
tT4	total thyroxine
TSH	thyroid-stimulating hormone
TSHst	TSH stimulation test
