# Supplementary material for: Development and internal validation of diagnostic prediction models using machine-learning algorithms in dogs with hypothyroidism
Source: Front Vet Sci. 2023 Dec 19;10:1292988. doi: 10.3389/fvets.2023.1292988 (PMC10758480; doi:10.3389/fvets.2023.1292988)
Supplement: Supplementary file 1 [file Table_1.DOCX]

| **Variable** | **Total** | | **Hypothyroid (n=82)** | | **Euthyroid (n=233)** | |  |
| --- | --- | --- | --- | --- | --- | --- | --- |
|  | **n.missing** | **%.missing** | **n.missing** | **%.missing** | **n.missing** | **%.missing** | |
| Hypothyroidism (yes/no) | 0 | 0 | 0 | 0 | 0 | 0 |  |
| Breed (FCI classification) | 0 | 0 | 0 | 0 | 0 | 0 |  |
| Gender | 0 | 0 | 0 | 0 | 0 | 0 |  |
| Age | 0 | 0 | 0 | 0 | 0 | 0 |  |
| Weight (Kg) | 0 | 0 | 0 | 0 | 0 | 0 |  |
| Serum total thyroxine (tT4) concentration | 0 | 0 | 0 | 0 | 0 | 0 |  |
| Serum thyroid-stimulating hormone (TSH) concentration | 0 | 0 | 0 | 0 | 0 | 0 |  |
| Asthenia | 0 | 0 | 0 | 0 | 0 | 0 |  |
| Lethargy/Depression | 0 | 0 | 0 | 0 | 0 | 0 |  |
| Polyuria and polydipsia | 0 | 0 | 0 | 0 | 0 | 0 |  |
| Obesity | 0 | 0 | 0 | 0 | 0 | 0 |  |
| Alopecia | 0 | 0 | 0 | 0 | 0 | 0 |  |
| Dermatopathy | 0 | 0 | 0 | 0 | 0 | 0 |  |
| Neurological alterations | 0 | 0 | 0 | 0 | 0 | 0 |  |
| Appetite | 1 | 0.3 | 0 | 0 | 1 | 0.4 |  |
| Serum creatinine concentration | 8 | 2.5 | 1 | 1.2 | 7 | 3.0 |  |
| Hematocrit | 8 | 2.5 | 1 | 1.2 | 7 | 3.0 |  |
| Serum alanine aminotransferase (ALT) activity | 9 | 2.9 | 2 | 2.4 | 7 | 3.0 |  |
| Hemoglobin | 9 | 2.9 | 1 | 1.2 | 8 | 3.4 |  |
| Red blood cells | 9 | 2.9 | 1 | 1.2 | 8 | 3.4 |  |
| Mean corpuscular volume (MCV) | 9 | 2.9 | 1 | 1.2 | 8 | 3.4 |  |
| Serum cholesterol concentration | 12 | 3.8 | 1 | 1.2 | 11 | 4.7 |  |
| Mean corpuscular hemoglobin concentration (MCHC) | 12 | 3.8 | 1 | 1.2 | 11 | 4.7 |  |
| Serum aspartate aminotransferase (AST) activity | 17 | 5.4 | 4 | 4.9 | 13 | 5.6 |  |
| Serum triglycerides concentration | 171 | 54.3 | 37 | 45.1 | 134 | 57.5 |  |
| Urine specific gravity (USG) | 205 | 65.1 | 59 | 71.9 | 146 | 62.7 |  |
| Serum tT4 post TSH | 209 | 66.4 | 43 | 52.4 | 166 | 71.2 |  |
| tT4 post TSH - % increase from baseline | 209 | 66.4 | 43 | 52.4 | 166 | 71.2 |  |
| Body Condition Score | 230 | 73.0 | 60 | 73.2 | 170 | 72.7 |  |
| Urine Protein-to-Creatinine Ratio | 250 | 79.4 | 69 | 84.1 | 181 | 77.7 |  |

**Table S1** Results of the analysis of missing data

**Table S2** Results of the analysis of correlation of the quantitative variables

| Variable | Age | | Weight | | T4 (nmol/L) | | T4 (µg/dL) | | TSH (ng/mL) | | Cholesterol | | Creatinine | | ALT | | Hgb | | HCT | | RBC | | MCV | | MCHC | |
| --- | --- | --- | --- | --- | --- | --- | --- | --- | --- | --- | --- | --- | --- | --- | --- | --- | --- | --- | --- | --- | --- | --- | --- | --- | --- | --- |
| Age | 1 |  |  |  |  |  |  |  |  |  |  |  |  |  |  |  |  |  |  |  |  |  |  |  |  |  |
| Weight | -0.0957 |  | 1 |  |  |  |  |  |  |  |  |  |  |  |  |  |  |  |  |  |  |  |  |  |  |  |
| T4 (nmol/L) | 0.0003 |  | -0.1513 | * | 1 |  |  |  |  |  |  |  |  |  |  |  |  |  |  |  |  |  |  |  |  |  |
| T4 (µg/dL) | 0.0003 |  | -0.1513 | * | 1.0000 | *** | 1 |  |  |  |  |  |  |  |  |  |  |  |  |  |  |  |  |  |  |  |
| TSH (ng/mL) | -0.0795 |  | -0.0099 |  | -0.3690 | *** | -0.3690 | *** | 1 |  |  |  |  |  |  |  |  |  |  |  |  |  |  |  |  |  |
| Cholesterol | -0.0862 |  | 0.1111 | . | -0.4059 | *** | -0.4059 | *** | 0.3130 | *** | 1 |  |  |  |  |  |  |  |  |  |  |  |  |  |  |  |
| Creatinine | -0.0077 |  | 0.1902 | ** | -0.1480 | * | -0.1480 | * | 0.1565 | ** | 0.0859 |  | 1 |  |  |  |  |  |  |  |  |  |  |  |  |  |
| ALT | 0.1192 | * | -0.1487 | * | -0.0087 |  | -0.0087 |  | -0.0065 |  | 0.0721 |  | -0.0971 |  | 1 |  |  |  |  |  |  |  |  |  |  |  |
| Hgb | -0.0643 |  | -0.1450 | * | 0.3445 | *** | 0.3445 | *** | -0.2203 | *** | -0.2073 | *** | -0.2145 | *** | 0.0058 |  | 1 |  |  |  |  |  |  |  |  |  |
| HCT | -0.0684 |  | -0.1565 | ** | 0.3638 | *** | 0.3638 | *** | -0.2325 | *** | -0.2029 | *** | -0.2052 | *** | 0.0779 |  | 0.8664 | *** | 1 |  |  |  |  |  |  |  |
| RBC | -0.0745 |  | -0.1055 | . | 0.3482 | *** | 0.3482 | *** | -0.2268 | *** | -0.2323 | *** | -0.1969 | *** | 0.0406 |  | 0.8136 | *** | 0.9182 | *** | 1 |  |  |  |  |  |
| MCV | 0.0266 |  | -0.1082 | . | 0.0675 |  | 0.0675 |  | -0.0076 |  | 0.0683 |  | 0.0472 |  | 0.1218 | * | 0.1381 | * | 0.2019 | *** | -0.1173 | * | 1 |  |  |  |
| MCHC | 0.0309 |  | 0.0375 |  | 0.0821 |  | 0.0821 |  | -0.0317 |  | -0.0467 |  | 0.0026 |  | -0.0670 |  | 0.2211 | *** | -0.0621 |  | 0.0192 |  | -0.1776 | ** | 1 |  |
